# Cognitive remediation therapy (CRT) as a pretreatment intervention for adolescents with anorexia nervosa during medical hospitalization: a pilot randomized controlled trial protocol

**DOI:** 10.1186/s40814-018-0277-5

**Published:** 2018-06-25

**Authors:** C. Alix Timko, Tiffanie J. Goulazian, Kathleen Kara Fitzpatrick, Daniel Rodriguez

**Affiliations:** 1 Department of Psychiatry, Perelman School of Medicine, University of Pennsylvania, Philadelphia, PA 19104 USA; 2 Department of Child and Adolescent Psychiatry and Behavioral Sciences, Children’s Hospital of Philadelphia, Philadelphia, PA 19104 USA; 3 Psychiatry and Behavioral Sciences, Stanford Children’s Hospital, Stanford, CA USA; 4 Department of Urban Public Health and Nutrition, La Salle University, Philadelphia, PA 19141 USA

**Keywords:** Cognitive remediation therapy, Anorexia nervosa, RCT, Adolescents, Mixed methods modeling

## Abstract

**Background:**

Anorexia nervosa (AN) is a severe psychiatric condition characterized by low body weight, fear of weight gain/becoming fat and/or behavior that interferes with weight gain, and body disturbance. Though there have been recent advances in the treatment of AN, there continues to be an urgent need to increase treatment options. Cognitive remediation therapy (CRT) has been successfully used as an adjunctive treatment for individuals with AN. In this study, we pilot the use of CRT plus an innovative parent involvement component as a pre-treatment intervention on a medical unit. We hypothesize that adding CRT with parent involvement to a standard hospital stay is feasible, acceptable by patients and staff, and may improve treatment outcomes post-hospitalization.

**Methods/design:**

This is a pilot randomized controlled trial with three arms. Participants are adolescents aged 12–18 with AN; 60 participants will be included. They are randomized into one of three groups: treatment as usual (TAU, standard care at Children’s Hospital of Philadelphia), CRT + contact control (known as “Family Fun Time”), and CRT + Teach the Parent. Intervention will occur on an inpatient basis. Follow-up will be outpatient and will continue until 6 months post-discharge. Psychosocial, neurocognitive, and behavioral measures will be collected throughout the study, and group differences will be evaluated at 4 weeks, 3 months, and 6 months post-discharge. The study will take place at The Children’s Hospital of Philadelphia.

**Discussion:**

This pilot randomized controlled trial will inform feasibility of the integration of a pre-treatment intervention into a medical hospital stay for AN. We will assess recruitment procedures, treatment administration, and participant retention. Finally, a comprehensive assessment battery will be evaluated. Secondary goals are to conduct a preliminary evaluation of whether or not CRT with parent involvement increases rate of weight gain and treatment engagement and decreases parental accommodation of symptoms post-discharge. If successful, this pilot study will inform a larger controlled trial fully powered to examine the secondary goals.

**Trial registration:**

ClinicalTrials.gov Identifier NCT02883413

## Background

Anorexia nervosa (AN) is a serious eating disorder characterized by restriction of food intake and reduction of body weight below what is healthy or expected for someone of the same age and height. Individuals with AN typically endorse fear or weight gain/getting fat or engage in behavior that can interfere with weight gain. Finally, those with AN can experience body dissatisfaction, disturbance in their experience of their body, or exhibit anosognosia. With the highest mortality rate of all psychiatric disorders [1, 2], AN is associated with significant morbidity [1] (including psychiatric co-morbidities and severe medical complications, i.e., cardiac complications, bone loss, electrolyte imbalances). Age of onset is typically during adolescence [3]; it significantly alters patterns of healthy growth and development and can impact the functioning of the entire family. Severe starvation during the critical period of adolescence has the potential to affect all developing systems, especially brain development [4–6]. Results from longitudinal studies of brain function in adults with AN indicate that sufferers may have long-term difficulties with executive functioning and have reduced cognitive and behavioral flexibility (e.g., perseveration, extreme perfectionism, difficulties in learning new behaviors) that reflects underlying neurocognitive inefficiencies [7, 8]. These inefficiencies are not deficits per se, as they are not usually clinically significant (in that they are rarely two standard deviations below expected performance for age); however, they are often areas of significant personal weakness in an overall cognitive profile. In this way, observed neurocognitive inefficiencies are intrapersonal deficits in executive functioning that could be indicative of underlying traits leading to a vulnerability to developing AN [9]. Cognitive inefficiencies could also be the result of starvation during a critical period of brain development or a marker for those who are likely to have a more chronic course of the disorder [10].

To date, there is only one intervention with substantial empirical support for the treatment of AN in adolescents: family-based treatment (FBT [11, 12]). In FBT, parents are essential to treatment and are charged with the task of re-nourishing their starving child. The effectiveness of FBT has established the importance of the family in treatment and empowered parents to play an active role in treatment [11, 13, 14]. The hypothesized mechanism of action in FBT is an increase in parental empowerment and a reduction in symptom accommodation [13]. However, FBT is not effective for all families and there is a significant portion of adolescents who do not reach remission in this treatment [12]. Recently, there has been an increasing focus on using our understanding of the neurobiology of AN to inform the development of or augmentation of existing treatments [15, 16]. Cognitive remediation therapy (CRT), an adjunctive treatment that focuses on neurocognitive inefficiencies, has growing support as being beneficial for individuals with AN [17, 18].

With its roots in neurology and neurosurgery, CRT was first developed in the early twentieth century to address cognitive impairments that occurred as a result of head injuries during WWI [19]. CRT can be conceptualized as a behavioral intervention that focuses on an individual’s cognitive skills. The expectation is that by improving cognitive functioning, an individual’s ability to manage daily life would be enhanced. Since its development, CRT has been used primarily for individuals with traumatic brain injury (TBI) or those who have suffered a stroke. Overall, the evidence for CRT as an adjunctive treatment for TBI is robust [20–23]. CRT can reduce memory failure, reduce anxiety, improve self-esteem and sense of self, and improve social relationships. CRT has been adapted for use in populations with psychiatric diagnoses, including schizophrenia and related disorders [24–26] and depression [27, 28]. There is a growing body of evidence indicating that it can be useful in the treatment of AN [29].

CRT is flexible in its implementation, as tasks included in a typical CRT intervention session can be altered to fit specific symptom profiles. A variety of tasks are presented to individuals, with each successive session requiring increasingly complex mental processes. Examples of tasks include geometric figures, illusions, reversing sequences of numbers and letters, completing sorting tasks wherein the rules unexpectedly change, and finding various routes on a map [30]. Regardless of the tasks used, CRT focuses on the development of meta-cognition—teaching individuals to think about how they think [17]. Most importantly, CRT focuses on process instead of outcome. This is especially salient for individuals with AN as CRT guides them away from a perfection-based or task-oriented perspective. Instead of focusing on whether a task was accurately completed, individuals in CRT are asked to think about *how* they solved a puzzle, (i.e., reflect on their thought processes and identify the steps used in solving that particular problem or puzzle).

### Why CRT for anorexia nervosa?

Cognitive flexibility is a trait and state variable hypothesized to be a risk and maintenance factor for AN. It encompasses a number of higher order executive functions, including set-shifting. Set-shifting is reflected in the ability to flexibly switch tasks, change mindsets, to rapidly adapt to new situations, and to respond to new contingencies in the environment [31]. Difficulties in set-shifting have been reliably observed in adults with AN when ill [32]. Improvements in set-shifting are often observed once recovered; however, women who are weight restored typically continue to display neurocognitive inefficiencies in relation to healthy controls [8]. Interestingly, siblings and parents of individuals with AN also have difficulties with set-shifting—leading to its hypothesized role as an endophenotype [7, 33]. Individuals with AN are also hypothesized to have weak central coherence, which likely persists past the ill state [34–38]. Central coherence is an information processing style that refers to global processing, i.e., the tendency to see the gestalt or big picture [39]. Individuals with AN have weak central coherence, that is, they tend to struggle to see the big picture and hyper-focus on details [36].

Together, difficulties in set-shifting and weak central coherence can contribute to cognitive inflexibility, a thinking style commonly observed in individuals with AN; they may reinforce to perfectionistic or compulsive behavior (both of which are risk factors for AN) [34]. For example, difficulties in set-shifting can be observed in the presence of rigid rules and behaviors surrounding food, body shape and size, need for control, and an unwillingness to change. Clinically, this can present as continued difficulties with increasing caloric intake or with eating a flexible variety of food. Weak central coherence is evidenced by difficulties in seeing the long-term goals of treatment and hyper-focusing on details of the treatment plan. Finally, our experience has been that difficulties in set-shifting affect the treatment and recovery process by reducing the likelihood that patients will seek out and remain in treatment or benefit from skills and therapeutic techniques that encourage change.

CRT for AN was developed to target observed difficulties in set-shifting and in central coherence [30]. CRT can improve brain function by exercising and increasing connections in the brain leading to greater flexibility with set-shifting and increases in global processing. Though not sufficient for a standalone treatment, CRT has been successfully used as an adjunctive approach. Initially developed to be used in an individual format with adults, the use of CRT was refined and further developed as a result of positive outcomes in case studies and early trials. It is flexible and modifiable: It can be delivered in individual, family, and/or group formats [40–45]. It has been shown to increase cognitive flexibility in adult and adolescent patients with AN [18, 44–46].

For adolescents with AN, CRT is generally well received and can result in increased motivation to engage in treatment [45]. Parents report feeling that the rationale behind CRT helps them understand their child’s behavioral difficulties [47]. In previous research, a family based form of CRT for adolescents with AN facilitated parents’ ability to recognize their own and their child’s thinking style (i.e., cognitive inflexibility) and helped the family to develop strategies to cope with these thinking styles [45]. Overall, CRT is generally well received by patients and parents, may increase treatment retention, and has a demonstrated impact on executive functioning [17]. Despite preliminary positive findings, research on CRT in adolescents is still limited [18]. The dearth of research in this area is a significant gap in our knowledge regarding how best to apply CRT. As such, the purpose of this pilot study is to test the feasibility of the use of CRT as a pre-treatment intervention during medical stabilization for AN.

### Rationale for CRT as an in-hospital, pre-treatment intervention

Adolescents with AN who present for treatment for the first time often need initial hospitalization for medical stabilization. CRT has been successfully developed for adolescents and for administration during a hospital stay [47]. Our rationale for integrating CRT during hospitalization for medical stabilization is that it will positively influence outcomes via increasing treatment engagement *post-discharge*. Given that CRT does not focus on eating disorder symptoms, we hypothesize that CRT may provide a non-threatening introduction to a therapeutic relationship, thereby increasing the adolescent’s willingness to engage in treatment once discharged. As family is important in the treatment of AN, we have included a parent component to our version of CRT. Adolescents will be asked to conduct modified versions of CRT sessions with their parents. We hypothesize that when adolescents conduct CRT with their parents, it will increase their parents’ understanding of their child’s thinking style. We further hypothesize that this understanding will translate into a greater understanding of the signs of when their child was having difficulty in areas such as re-nourishment or behavioral flexibility post-discharge. Our hope is that this improved understanding will provide parents with additional support for reducing accommodation of symptoms—with the potential of leading to greater rate of weight gain and engagement in treatment.

By having adolescents teach their parents CRT, we are drawing on the principles of “train the trainer” models and are strengthening adolescents’ abilities to shift gears and think globally. In train-the-trainer programs, a health care provider uses disease specific educational materials to train other providers who, in turn, disseminate the information. In our approach, the study interventionist trains the adolescent in greater meta-cognitive awareness. The adolescent then, in turn, teaches this to their parent. This approach has a number of benefits. In addition to providing the parent with insight into what their child is learning about the child’s thinking styles, parents have the opportunity to learn a bit about their own. It also provides the adolescent with an opportunity to practice what they are learning during CRT sessions and to consolidate any gains they have made. We know from prior research that patients with AN find the time spent in CRT appealing, as it does not focus on the eating disorder or discuss challenges with eating in any way [46]. We anticipate parents will experience this positive effect as well. By providing time for the adolescent and parent to interact around non-eating disorder tasks, we believe it will foster bonding and healthier, non-disease specific family interactions and processes.

Based on this rationale, we have shifted our outcome variables of interest for our pilot study using CRT as a pre-treatment intervention. Traditionally, the outcome variables for CRT are changes in cognitive flexibility. While important, we hypothesize that CRT may have more immediate positive impacts that will support its potential as a pre-treatment intervention [48]. We will examine whether CRT combined with a novel parental portion can positively impact behavior post-discharge. We hypothesize that CRT during a hospital stay will increase treatment engagement, increase rate of weight gain post-discharge, reduce symptom accommodation, and increase behavioral flexibility in adolescents and parents. As the site for this research is a medical unit with no psychosocial interventions, we opted to conduct a pilot and feasibility randomized control trial to pilot study procedures and refine the protocol prior. Doing so allows us to evaluate the feasibility (including burden for staff and parents and the acceptability of the treatment) of conducting CRT with a parent component on a medical unit and determine what changes may need to be made for a fully powered trial [49, 50].

### Objectives

#### Primary aims

Cognitive remediation therapy (CRT) with adolescents admitted to hospital for medical stabilization of AN is a pilot and feasibility randomized controlled trial. There are three conditions in this study: treatment as usual (TAU; control), CRT + Teach the Parent (CRT + TTP; experimental), and CRT + Family Fun Time (CRT + FFT; contact control). Interventions are outlined in Tables 1 and 2. The primary aim of the study is to determine whether or not CRT with a family component could be adapted for daily delivery during a hospital stay for medical stabilization. As this is the first time that intervention research for AN is occurring on the medical floor at Children’s Hospital of Philadelphia (CHOP), we also sought to pilot recruitment, randomization procedures, and assessment batteries; to establish recruitment rates and retention during follow-up, treatment administration, and evaluate the feasibility (including burden to staff and parents and acceptability of treatment) of conducting CRT with a parent component on a medical unit.

**Table 1:** Summary of tasks included in CRT for both conditions

CRT Task	Session number	Description	Target
Introduction to CRT and to either Family Fun Time or Teach the Parents	1	Review of the purpose of CRT and informing family of which condition they have been randomized to. Family and participant receive handout summarizing rationale of CRT, and receive appropriate manual outlining CRT sessions.	
Alternate uses	1–8	Generate atypical uses for everyday objects	SS
Geometric figure	1–8	Adolescents describe geometric figures and have the interventionist draw the figure based on cues, then discuss strategy for how adolescent approached giving instructions	CC
Hand task	1–8	Repetitive, motor skills task involving use of the dominant as well as non-dominant hand and fingers	SS
Illusion task	1–8	Introduction and discussion of increasingly difficult illusions	SS and CC
Infinity signs	1–8	Drawing infinity signs with dominant, inferior, and both hands	*
Stroop	1–8	Participant reads words on a sheet of paper while switching back and for the between the color the word is printed in and reading the word, an exercise of set-shifting that increases in intensity over the CRT sessions.	SS
Switching attention	1–5 (6–8 optional)	Asking participant to switch their attention repeatedly back and forth between two different topic categories, such as movie titles and spices	SS
Main idea	1–8	Reading increasingly difficult text selections and summarizing the main idea in a short sentence	CC
Qbitz	1–8	Completion of a puzzle with small wood blocks designed to increase manual dexterity and encourage a variety of problem solving approaches.	CC and SS
Rebus	1–8	Visual or pictorial representations of common words, phrases, or idioms. Uses verbal and perceptual skills.	SS and CC
Set	1–6	Creation of sets using features of objects that are similar in specific ways	SS
Map	7–8	Encourages participants find different ways of navigating a map, utilizing verbal and spatial abilities, organizational skills, combining elements of set-shifting and central coherence	CC and SS
Homework review	2–8	Review tasks completed and link to what has been happening in CRT sessions	
Review of parent session	3, 5, 7	Review tasks that have been completed, and review the overall process of TTP or FFT	

*SS* set-shifting, *CC* central coherence

*Motor task in which adolescents can, in a very short period, see how practice can improve performance. It has an element of shifting as the task must be completed with each hand. This task also helps to teach implicit learning and highlight the importance of non-verbal learning and fluidity

**Table 2:** Train the parent and Family Fun Time session-specific tasks

Session no.	Train the Parent (TTP) tasks	Family Fun Time (FFT) tasks
1	Introduction to CRT + TTP Illusion task Infinity signs Stroop Geometric figure	Introduction to Family Fun Time I Learn About You—You Learn About Me (favorite TV show) Coloring: color and switch Table topics Family choice
2	Review and update Illusion task Infinity signs Geometric figure Main idea	Review and update I Learn About You—You Learn About Me (listen to favorite song) Coloring: color and switch Table topics Family choice
3	Review and update Illusion task Infinity signs Main idea Qbitz	Review and update Mad Libs Table topics Sudoku Family choice
4	Review and update Illusion task Infinity signs Hand task Qbitz	Review and update Table topics Jenga *or* Apples to Apples UNO *or* Sudoku Coloring *or* Mad Libs Family Choice

#### Secondary aims

The secondary aims are to gather preliminary evidence as to whether or not CRT with parent involvement reduces symptom accommodation by parents, increases adolescents’ willingness to engage in treatment, and increases rate of weight gain *post-hospitalization*. As a traditional outcome of CRT, changes in cognitive flexibility will also be assessed. We anticipate adolescents in the CRT conditions will have greater treatment engagement upon discharge and will have an increase in cognitive flexibility at 6-month follow-up. We further hypothesize that parents in the TTP condition will demonstrate less accommodation of symptoms at 3- and 6-months post-discharge. Finally, we hypothesize that adolescents in the TTP condition will have greater rate of weight gain post-discharge than adolescents in the FFT condition and that adolescents in both CRT conditions will have greater rate of weight gain in comparison with the adolescents in the treatment as usual condition. Given that this is a pilot study, we know that full exploration of these hypotheses will be limited. However, these are the hypotheses we plan on testing in a fully powered trial.

## Methods/design

### Ethical considerations

The Institutional Review Board (IRB) of Children’s Hospital of Philadelphia reviewed and approved all study materials, consent forms, and protocols. The IRB will approve all amendments and will be informed of any reportable events throughout the trial. Any modifications to the initial protocol will be described in detail in a manuscript describing the implementation of the study protocol. The principal investigator, research staff, and study interventionists have completed CITI training, including additional staff training specific to Children’s Hospital of Philadelphia. Written assent is obtained from all adolescent participants; parents provide written informed consent for their child and themselves. Methods for collecting, recording, and reporting of data are designed to ensure the privacy, health, and welfare of research subjects both during, and after, the study.

### Setting

This pilot and feasibility study will take place at Children’s Hospital of Philadelphia. Adolescents with malnutrition are admitted to a general medical unit and placed on a standard nutritional rehabilitation protocol. The protocol is described in detail elsewhere [51]. In short, adolescents with AN are typically admitted for medical complications due to malnutrition. They are managed by the Eating Disorder Assessment and Treatment program, an interdisciplinary team that includes medical staff, behavioral health, nutrition, nursing, etc. Importantly, parents are included as part of the treatment team. Parents are encouraged to be with their child as much as they can, and many will stay overnight with their child. Parents are included in treatment. In addition to medically stabilizing a child and beginning the nutritional rehabilitation process, time in hospital is used to educate families about the eating disorder and the child’s nutritional needs when at home (i.e., account for hyper metabolism during nutritional rehabilitation). Psychological services are limited to diagnostic assessment/clarification and communication with parents, but no specific psychosocial interventions are offered during hospitalization.

The eating disorder treatment program at CHOP is family-based. As noted above, parents are very involved in their child’s hospital stay. The inherent inclusion of parents in treatment makes CHOP an ideal place to test the feasibly of adding CRT with a parent involvement component to treatment while in hospital. Patients in the program are overwhelmingly female, white, and range in age from approximately 6–23. Most meet criteria for severe malnourishment, and two thirds meet diagnostic criteria for AN [52].

### Participants

Participants are male and female adolescents aged 12–18 who present in hospital for treatment of a restrictive eating disorder and at least one caregiver. Only adolescents who have not had prior multidisciplinary treatment for an eating disorder will be included. Inclusion and exclusion criteria are summarized in Table 3.^1^ The total enrollment is anticipated to be 60 adolescents.

**Table 3:** Inclusion and exclusion criteria

Inclusion criteria	Exclusion criteria
• Adolescent is between 12 and 18 years of age and living at home with caregiver(s) • Adolescent meets diagnostic criteria for anorexia nervosa (either restricting or binge/purge subtype), or sub-threshold anorexia nervosa according to the DSM-5 criteria • Parent or primary caregiver is willing to participate in CRT + TTP or CRT + FFT sessions (if randomized to either condition) • Consent of all family members who will be participating in treatment • The adolescent is not currently receiving outpatient treatment for the eating disorder (i.e., their first presentation at CHOP)	• Caregiver or adolescent with a co-morbid diagnosis of psychotic disorder, substance dependence, substance abuse, or bi-polar disorder • Caregiver or adolescent with diagnosis of intellectual disability, developmental delay, or autism spectrum disorder • Adolescent with a diagnosis of feeding or eating concerns not elsewhere classified with primary symptoms of bingeing and purging, bingeing without compensatory behaviors, or spitting food with restricting patterns. • Adolescent with diagnosis of avoidant/restrictive food intake disorder • Adolescent or caregiver with acute suicide risk • Concurrent psychosocial treatment for another condition • Adolescent or parent (who will be participating) not fluent in English

### Study design

**Fig. 1:**
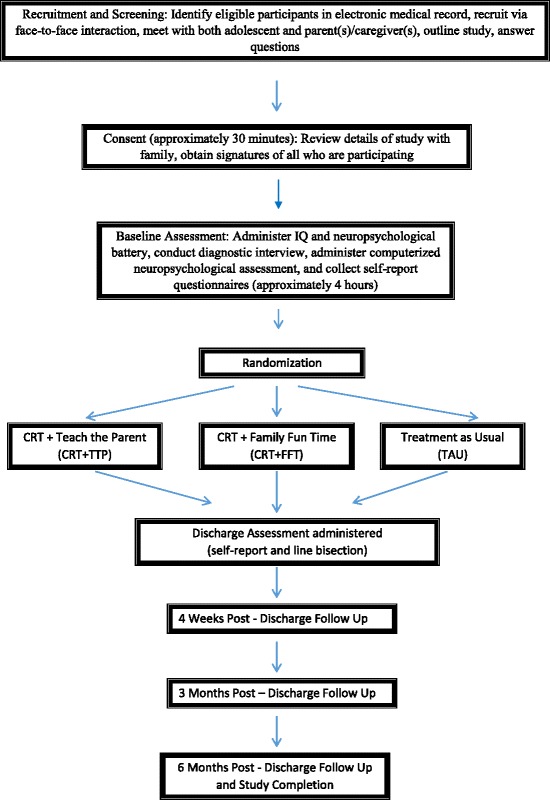
Study flowchart/timetable for data collection

This is a pilot and feasibility study for a randomized controlled trial that is prospective in nature and pragmatic in its approach. The study uses a randomized, three-group design (control, experimental, and contact control) with five assessment time points: baseline (upon admission to hospital for medical stabilization), pre-discharge (the day of, or the day before they leave the hospital), 4 weeks post-discharge, 3 months post-discharge, and 6 months post-discharge. Figure 1 outlines the study flowchart and timetable for data collection.

After consent is obtained and baseline assessment is completed, participants are randomized to one of three groups: CHOP’s standard care (treatment as usual; TAU), CRT + contact control (known as “Family Fun Time”), and CRT + Teach the Parent. Those randomized to one of the CRT conditions will receive individual sessions of CRT daily (excluding weekends) while in hospital. Follow-up will be naturalistic. All attempts will be made to follow participants whether they enter outpatient treatment or are referred to a higher level of care. Psychosocial, neurocognitive, medical, and behavioral measures will be collected throughout the study. Group differences will be evaluated at each of the five assessment time points.

### Recruitment and screening procedures

Potential participants are identified during the first 48–72 h after hospitalization for medical stabilization due to malnutrition. Initial screening occurs via review of the electronic medical record. Participants who appear eligible are approached by research assistants and the study and eligibility criteria are described. They are provided with information about the clinical trial (there are currently no other intervention studies and alternatives to participation is treatment as usual), are provided with detailed information regarding the study logistics, and are able to ask any questions. If interested, parents provide consent for themselves and the child provides assent. Adolescents who are 18 years of age provide consent for their participation. Consent ideally occurs within 12–24 h of being informed of the trial and expressing an interest in participating. Study procedures do not take place during meal times, family time, or while visitors are present.

### Description of investigational intervention

The principles of cognitive remediation therapy are the same across studies; however, CRT protocols often vary in terms of the tasks used. This trial uses a modified version of an adolescent manual developed by K.K. Fitzpatrick [47]. The CRT tasks included in our manual are delineated in Table 1. CRT sessions occur daily (excluding weekends) for 45–60 min every day while adolescents are in hospital, for up to 8 days. We have set an a priori definition of “treatment completion” as six sessions of CRT. Treatment completion for parent involvement component was set a priori at three sessions of either TTP or FFT. Though the median length of stay in hospital is 10 days, it is possible that adolescents may discharge before completing all sessions of CRT. These adolescents will continue to complete all study procedures (i.e., follow-up) but will not be considered treatment completers for feasibility assessment (what percentage of adolescents complete CRT) or data analysis (secondary goals).

Study interventionists include the PI or a graduate student under the PIs supervision. K.K. Fitzpatrick provided initial training in CRT, and will provide supervision and feedback to the PI throughout the study duration. CRT sessions will be audio-recorded and coded for adherence to the manual. Should a study interventionist deviate from the manual (e.g., do tasks out of order), this will be noted in the data set. The interventionist will be informed and further training provided to ensure adherence.

CRT sessions are administered every weekday while the adolescent is in hospital, are conducted in an individual format, and tasks are delivered in a standard order to ensure consistency. Table 1 lists all tasks in the standardized order in which they are presented each session. Table 1 also notes the session in which the task occurs; most tasks are administered in each session. It is possible that not all tasks will be able to be administered due to time constraints or individual variables (i.e., hand flip if the adolescent has an IV). At the end of the pilot study, we will re-evaluate the tasks and determine if all should be included a final manual.

Each task has a variety of levels that the adolescent moves through progressively. A particular level of task is repeated in subsequent sessions if the adolescent has difficulty with it. They move to the next level of that task only when they have mastered the current level. For example, each CRT session uses a progressively more difficult version of the Stroop task (e.g., BIG/little—reading the word versus saying “big” if written in uppercase and “little” if written in lower case versus the same type of task using SMALL/large). If the adolescent has difficulty with the Stroop task, they complete the same level of the task until they have mastered it. In the next session they move to the next level of the task. Thus, it is possible that an adolescent can progress through a new level of task each session, yet repeat the same level of a task for a different session (note, the task level is recorded by the interventionist each session).

After each task, the adolescent and study interventionist discuss the process by which the adolescent completed the task. Meta-cognition is fostered and the adolescent is asked to think about real life examples that mirror what was done in session. A series of standardized prompts are used to the guide the discussion, though not all prompts may be necessary in order to generate a discussion. The eating disorder is not discussed unless touched upon by the adolescent. When appropriate, connections to in hospital behavior can be drawn. Between sessions, homework and behavioral tasks are assigned to participants so they can practice what they are learning in CRT. Starting with session 2, the session begins with homework review prior to the CRT tasks.

### Randomized groups

#### Cognitive remediation therapy and Teach the Parent (TTP)

This parental involvement addition to CRT is designed to increase parents’ understanding of their adolescent’s thinking styles and reinforce what the adolescent learns about their own style. We hypothesize that by including parents, they will be more likely to challenge eating disorder behaviors and be less likely to accommodate behavioral symptoms of the eating disorder (e.g., make something low-fat for dinner because the adolescent will not be as upset and agrees to eat the low calorie food). In TTP, adolescents receive their own treatment manual to use during their CRT sessions with their parents. Adolescents do tasks with their parents that they have already done with study staff. Adolescents are instructed to explain the rationale for CRT, what they have learned, and do four tasks with their parents. Table 2 lists the tasks done during each TTP session. The family is instructed to refrain from speaking about the eating disorder during CRT. TTP sessions occur up to four times (every other CRT session). A research assistant will be on hand to observe the session and ensure that the adolescent presents at least two tasks to parents. However, the research assistant will not engage with the family.

#### Cognitive remediation therapy and contact control (Family Fun Time, FFT)

In order to assess for any non-specific effects of spending non-eating disorder driven time with family, adolescents in the CRT + contact control condition will be asked to spend 3–4 sessions with their parents engaging in fun activities (games, coloring, trivia). We refer to this condition as CRT + Family Fun Time (CRT + FFT). The family is provided with the rationale that engaging in imaginative and fun play can help to stimulate brain development. Tasks were chosen to be engaging and superficially relevant to CRT (see Table 2 for a list of session-specific TTP and FFT tasks). As with TTP, adolescents are provided with an FFT manual and are asked to guide their parent through the session. Specific tasks are laid out each session and adolescents are provided with the necessary materials. As with TTP, the family is asked to refrain from eating disorder talk. Research assistants will be on-hand to observe interactions but will not engage with the family.

#### Treatment at usual

Adolescents in this condition will not receive any experimental intervention. They will have a standard hospital stay with all normal contact with a multidisciplinary team of health professionals: attending physician, psychologist, social worker, and dietician. Medical stabilization for adolescents with AN at CHOP has been described elsewhere [51].

### Blinding and randomization

The adolescent and at least one parent are required to participate in the study. After baseline assessment is completed (see Table 4 for measurement schedule according to the SPIRIT guidelines [53, 54]), the adolescent is randomized to a condition. Research assistants completing the baseline assessment are therefore blind to the condition at baseline.

**Table 4:** Schedule of enrollment, intervention, and assessment

Study period													
	Enrollment	Baseline		Randomization	CRT sessions/intervention	Discharge		4 weeks post-discharge		3 months post-discharge		6 months post-discharge	
Timepoint	− t_1_	T_1_		T_1a_	Up to 8 sessions No assessment	T_2_		T_3_		T4		T5	
Enrollment													
Eligibility screen	X												
Informed consent	X												
Randomization				X									
Intervention													
CRT + TTP					X								
CRT + FFT					X								
TAU					X								
Assessments													
		*Adol.*	*Par.*			*Adol.*	*Par.*	*Adol.*	*Par.*	*Adol.*	*Par.*	*Adol.*	*Par.*
*Diagnostic/symptom tracking*													
EDE [55]		X								X			
EDE-Q [70]		X						X		X		X	
EDE-SF [52]		X				X		X		X		X	
ABOS [71]			X						X		X		X
BASC-2[72]		X	X					X	X	X	X	X	X
Weight and height								X		X		X	
*Medical chart review*													
Weight history		X											
Current weight		X				X		X		X		X	
Hormone levels		X								X		X	
Vital signs		X				X		X		X		X	
Daily caloric intake		X				X				X		X	
*Neuro-psychological*													
WASI (2 scale) [73]		X	X										
D-Kefs [74]		X	X							X	X		
BRIEF [75]		X	X					X	X	X	X	X	X
Hayling [76]		X	X							X	X		
Rey-O [77]		X	X							X	X		
Brixton [76]		X	X							X	X		
Line bisection [78]		X	X			X	X	X	X	X	X	X	X
PennCNB [79]		X	X									X	X
*Questionnaire*													
C/E [80]		X	X			X	X						
MSCARED [81]		X	X			X	X	X	X	X	X	X	X
EDQoL [82]		X				X		X		X		X	
DBS [83]		X				X		X		X		X	
CFS [84]		X	X			X	X			X	X	X	X
DFlex [85]		X	X			X	X	X	X	X	X	X	X
C-FOCI [86]		X				X				X		X	
YBC-EDS-SRQ [87]		X						X		X		X	
AFQ-Y [88]		X								X		X	
AAQ [89]			X						X		X		X
AESED [90]			X						X		X		X
BIS/BAS [91]		X	X					X	X	X	X	X	X
PAAQ [92]			X						X		X		X
DASS-21 [93]			X						X		X		X
FQ [94]			X						X		X		X
IES [95]			X						X		X		X

Note. *Adol.* adolescent, *Par.* parent, *TAU* treatment as usual, *CRT + TTP* cognitive remediation therapy + Teach the Parent, *CRT + FFT* cognitive remediation therapy + Family Fun Time, *EDE* Eating Disorder Examination, *EDE-Q* Eating Disorder Examination Questionnaire, *EDE-SF* Eating Disorder Examination Short Form, *ABOS* Anorectic Behavior Observation Scale, *BASC* Behavior Assessment System for Children-2, *WASI* Wechsler Abbreviated Scale of Intelligence, *D-Kefs* Delis-Kaplan Executive Functioning Scales (Trail Making, Verbal Fluency, Color-Word Interference, & Sorting), *BRIEF* Behavior Rating Inventory of Executive Function, *Rey-O* Rey-Osterrieth Complex Figure Test, *PennCNB* computerized neuropsychological battery, *C/E* Credibility/Expectancy Questionnaire, *MSCARED* Motivational Stages of Change for Adolescents Recovering from an Eating Disorder, *EDQoL* Eating Disorder Quality of Life, *DBS* Decisional Balance Scale, *CFS* Cognitive Flexibility Scale, *DFlex* Detail and Flexibility Questionnaire, *C-FOCI* Children’s Florida Obsessive Compulsive Inventory, *YBC-EDS-SRQ* Yale-Brown-Cornell Eating Disorders Self-report, *AFQ-Y* Action Fusion Questionnaire—Youth, *AAQ* Acceptance and Action Questionnaire, *AESED* Accommodation and Enabling Scale for Eating Disorders, *BIS/BAS* Behavioral Inhibition System and Behavioral Activation System Scales, *P-AAQ* Parental Acceptance and Action Questionnaire, *DASS-21* Depression Anxiety and Stress Scale-21, *FQ* Family Questionnaire, *IES* Impact of Events Scale

Randomization was obtained via block randomization using values from Research Randomizer, an online randomization program (http://www.randomizer.org/). An undergraduate research intern completed the randomization prior to recruitment starting, wrote the condition number on an index card, and sealed the index card in a security envelope. Envelopes are numbered consecutively. As participants consent to participate, they are assigned the next number and that envelope is pulled and put in their research chart. Once assessment is complete, the envelope is opened and the condition recorded in a password protected file.

The majority of follow-up assessments (T_2_, T_3_, and T_5_) are primarily questionnaire based. Families return at T_4_ to complete the neuropsychological battery. Research assistants who conduct the assessments at this time point are not specifically blinded to condition; however, they are also not informed of the family’s experimental condition. As research assistants open the randomization envelopes, they may independently recall a family’s condition. Data analysis (see below) will be blinded. The statistician and PI (DR and CAT, respectively) will not be aware of the coding for condition until after data analysis is complete. The study coordinator will code the data and be responsible for keeping the investigators blind.

### Measurement strategy

As this is a pilot and feasibly RCT, we chose a comprehensive measurement battery that closely resembles batteries used in prior research investigating set-shifting, central coherence, and CRT in individuals with AN. See Table 4 for all measures and the measurement schedule. A goal of the study was to evaluate the utility of the battery and streamline it for future research. Both parents and the adolescent complete a standard self-report questionnaire battery, neuropsychological assessment, and IQ assessment. In addition, the adolescents complete a semi-structured diagnostic interview to assess eating disorder symptoms (Eating Disorder Examination, EDE [55]). We included measures designed to assist us in evaluating our outcome variables. As neurocognitive inefficiencies are hypothesized to be endophenotypes [7, 37], we included IQ and neuropsychological assessment of both adolescent and parents. Baseline assessment is comprehensive and typically lasts a significant portion of the day. Families are informed of the length of the intake during the screening phase.

As the adolescents are in hospital, the baseline assessment can occur over multiple days. For adolescents, the neuropsychological assessment is prioritized, as we want to be able to assess for any impairment that may be present when acutely underweight. Randomization occurs once neuropsychological assessment is complete; it is possible that the EDE and self-report may not be completed until after randomization. Parent neuropsychological and IQ assessment is scheduled at the parents’ convenience throughout the week that their child is in hospital. If parents are not able to complete the IQ assessment at baseline, it can be administered at 3 months post-discharge as parental IQ is expected to be stable.

In order to facilitate participant retention and to reduce burden on families, all baseline assessments will take place while participant is in hospital. After consent is obtained, all participants will be given baseline questionnaires and the baseline assessment scheduled. The pre-discharge assessment questionnaires are given the day before or the day of discharge. Every effort will be made to collect both discharge questionnaires prior to the participant discharging. Follow-up assessment can be completed either on-line (via RedCap) or via pen and paper questionnaires mailed to families. When neuropsychological assessment needs to occur, we coordinate assessment with normal medical or behavioral health follow-up.

### Outcomes

The primary aim of the study is to evaluate the feasibility and pilot the procedures for an RCT of CRT with a family component delivered during a hospital stay for medical stabilization. Primary outcomes are as follows:

1. Recruitment rates (% of patients admitted who are eligible, % enrolled). Ideally, we will be able to recruit 30% of those eligible and at least four adolescents and their parents a month.
2. Completion rates (% of those enrolled who complete at least 6 sessions). Completion rates will vary based on the medically dictated hospital stay. As we have no control over when an adolescent will be discharged, completion rates will be combined with enrollment rates to determine how many participants would need to be enrolled in a future study (over-enrollment) to ensure the appropriate number of treatment completers.
3. Refusal rates and reasons why
4. Retention rate (% of those enrolled who completed all assessment points, rate of drop out over 6 months of follow-up). Ideally, we would have a dropout rate < 25%. As adolescents are discharging to various levels of care, we track dropout rates and determine if we have higher rates associated with certain levels of care (e.g., residential treatment).
5. Average length of time to complete measures

We will also pilot procedures and evaluate the following:

1. Ease of record forms (can the study staff use forms effectively and can the interventionist easily record data needed from each CRT session)
2. CRT task adaptations (e.g., are some tasks more difficult for adolescents, are there modifications that need to be made)
3. Staffing needs (are there enough staff to recruit/enroll/assess/and administer CRT?)
4. Acceptably of RCT to medical staff (evaluated through meetings with and feedback from staff (e.g., physicians, nurses, behavioral health)

In addition to piloting the study and assessing feasibility, our secondary goals are to examine relevant outcomes for a fully powered trial. We will examine the following:

1. Willingness of adolescent to engage in treatment post-discharge
2. Adolescent rate of weight gain post-discharge to 6 months
3. Parental accommodation in symptoms post-discharge (group differences and over time changes)
4. Changes in set-shifting or central coherence

### Data management and analysis plan

All study participants will receive a unique code that will be used for the duration of the study. This file linking this code to the participants will be stored in password-protected file in the PI’s research lab. All data will be entered into the database using this number. Upon completion of the study, the file linking the participant number to each participant will be destroyed. All data collected will be double scored and double entered into the database to ensure accuracy. The database will be coded. Once the list linking participant study identification numbers to specific participants is destroyed, the database will become de-identified. The data will be stored on a secure network drive that only the PI and team members have access to.

There will be no interim analysis conducted. Study results will be reported at national conferences and data will be submitted for publication.

We will assess treatment credibility and acceptability, track the number of adolescents who meet study criteria, the number approached for enrollment, the number enrolled, reasons why adolescents chose not to participate, dropout rate, and reasons for dropout (using the criteria set forth by DeJong and colleagues) [56]. These data (along with baseline and demographic characteristics) will be summarized by standard descriptive statistics (e.g. means and standard deviations for continuous variables such as age and percentages for categorical variables such as gender).

#### Efficacy analysis

Although this is a pilot study, we do want to explore whether or not there is a difference between groups. In order to determine differences between those who are randomized to TAU, CRT + FFT, or CRT + TTP, we will conduct a mixed multivariate analysis of variance (MANOVA) with five repeated measures (time: baseline, discharge, 4 weeks post-discharge, 3 months post-discharge, and 6 months post-discharge) as a within-subjects factor and treatment as a three-level between subjects’ factor. We will also evaluate the condition (treatment) × time interaction. We have three quantitative dependent variables: adolescent treatment engagement, post-treatment weight gain, and symptom accommodation. For initial analyses, we will focus on change pre- to 4-week post-discharge. Any post hoc comparisons will involve comparing subsequent assessments to baseline—as they will be simple planned comparisons. This will allow us to retain the most amount of power (i.e., to enable us to reject the false null hypothesis) [57]. We will also set alpha at .10, which will also increase power. Given these parameters, enrolling 60 adolescents would give us an N comparable to prior research using CRT [58].

In addition to the prior analyses, we will also assess developmental trajectories for rate of weight gain post-discharge, treatment engagement post-discharge, and symptom accommodation using latent growth curve modeling (LGCM) with one growth curve analysis for each variable. LGCM is a structural equation modeling method that models repeated observed measures (measured variables) on factors (latent variables) representing random effects [59]. In LGCM, a level factor is used to represent baseline and trend factors are used to represent rate of change across time. Latent variables define the form of rate of change across time (e.g., linear, quadratic, cubic). Factor loadings are fixed to define baseline level and trend. For a linear trend, the factor loadings are set so they increase uniformly with each unit increase in time. Given is a preliminary analysis, these results will allow us to better understand how change occurs over time. Instead of relying on probability values which subject us to increased type II error rates given the small sample size (i.e., low power), we will assess confidence intervals around our estimates to provide information regarding margins of error. All confidence intervals will be 90% confidence intervals given the small sample sizes.

#### Model fit criteria

The following suggested model fit criteria will be used to assess the fit of the latent growth curve model to the data: non-significant model chi-square, CFI above .95, RMSEA below .05–.08, and a SRMR value below .08 [60, 61].

#### Missing data

Every effort will be made to avoid missing data, particularly with respect to our follow-up data points. However, missing data is a common problem in longitudinal research, so it is important to be aware of sources and plan appropriate procedures for accommodating missing data [62]. Missing data can be broadly classified as missing at random or missing not at random and are either ignorable or not ignorable. To assess type of missing data, we will employ various tests for the effects of missing data on our sample (e.g., homogeneity of growth parameters across different patterns of missing data and pattern-mixture modeling). There are several methods for dealing with missing data including ignoring missing data and modeling with complete data only. However, this strategy can bias parameter and standard errors by utilizing smaller proportions of available data and depends on the assumption that data is MCAR [62]. An alternative involves data imputation with multiple imputation [63].

#### Power analysis

As this is a pilot study, the primary outcome is understanding the feasibility of implementing a large-scale trial. For the mixed MANOVA and using G*power 3.1.7 [64, 65] with three groups (*n* = 20 per group) and five repeated measures, using a 10% probability of a type I error (α = .10), and a medium effect (*f* = .25), the power achieved was .55.

To assess power in the LGCM, we conducted a series of Monte Carlo simulations with 1000 replications, to determine sample size for sufficient power to evaluate developmental trajectories of our primary and secondary outcome variables in a sample of 60 participants with AN. We used M*plus* 7.2 software for all simulations [66]. Population parameter estimates were derived using data from our prior longitudinal analyses. We concluded sufficient power only if the simulation resulted in parameter and standard error bias less than 10% and 15%, respectively, coverage close to 95% (i.e., confidence interval includes the population value), and power above .80 for the significant effects [66–68]. Power calculations were based on ability to detect small but meaningful effect sizes for intercept (baseline level) and trend (rate of change) factors [69]. The results supported low bias (< 10%), acceptable parameter coverage values for the intercept (.95) and trend (.944) factors, and sufficient power (>.95).

## Discussion

The current study is a pilot and feasibility study that examines the implementation of CRT on a medical unit in a large children’s hospital. The description of this protocol followed SPIRIT guidelines [53, 54]. CRT + TTP is compared to treatment as usual and a CRT + contact control group (CRT + FFT). As this is a pilot and feasibility study, we have specified the outcomes we would use in a larger, randomized clinical trial. We plan to focus on the viability of integrating CRT into clinical care, refine recruitment procedures and estimate recruitment rate for a larger trial, and pilot the feasibility of the assessment strategy. Inpatient hospitalization presents as a unique opportunity for us to examine our primary outcomes of interest and establish the feasibility of this type of intervention in the context of a medical unit. Scaling this study up for a larger trial will be feasible if CRT is able to be delivered with little interruption to clinical care, if study interventions are not perceived as burdensome to staff or families, and if participants are able to be successfully recruited and retained through the follow-up phase. By evaluating our rate of enrollment and follow-up and the % of treatment completers, we should be able to determine how long it would take to enroll enough treatment completers to have a fully powered trial. If it would take 3+ years to recruit enough participants from one site, we would need to consider if it feasible to scale up the study or if a multi-site study is needed. The secondary aims could help to inform this—particularly if we see trends in a positive direction. If there is no trend for a difference between TAU and the experimental groups on secondary outcomes and it would take a two-site study to complete, scaling up of this work may not be feasible.

The study intervention builds upon prior research on the use of CRT as an adjunctive treatment for AN. In particular, this study uses CRT as a pre-treatment intervention. While increases in cognitive flexibility are typically the primary outcome of CRT, we posit that CRT may be particularly useful in increasing willingness to engage in treatment upon discharge from the hospital. In addition to assessing stages of change, we are also examining the rate of weight gain when discharged.

Family-based treatment is the only empirically supported treatment for AN; one hypothesized mechanism of action is a reduction in parental accommodation of symptoms [13, 14]. We believe that by adding a parent involvement component to CRT, it will increase parents’ awareness of their child’s cognitive style and could contribute to a reduction in symptom accommodation post-discharge from the hospital. The “Teach the Parent” component of CRT added is a novel addition to current work being done utilizing CRT. This model is particularly suited to hospital stays as youth and their parents often have down time while in hospital. Conducting CRT with their parents allows children to practice skills they learned in their own CRT session, provides them with the time to interact with parents (without a focus on why they are in hospital), and helps to teach parents about their and their child’s cognitive flexibility. Our hope is that by increasing parental awareness of the process of their and their child’s thinking they will be able to persist in re-nourishment, despite the increasing intensity of symptoms that adolescents may experience once they are discharged and living at home.

As a considerable strength of this pilot study, the methods used (recruitment, assessment, randomization, intervention, and follow-up) are being piloted in an actual clinical, real-time hospital setting. There are no restraints on the type of treatment that adolescents will discharge to, although the majority of adolescents in CHOP’s program do enter outpatient treatment. In addition, details regarding the medical care that adolescents receive (TAU) have been detailed in a prior publication [51]. Results from this study will inform a larger clinical trial to determine the effectiveness of CRT as a pre-treatment intervention, particularly with the inclusion of a parental involvement component. Our outcomes (changes in willingness to engage in treatment, rate of weight gain, and parental accommodation of symptoms) are novel for CRT studies. Although not of primary interest, we are collecting additional data on cognitive flexibility and will track changes that occur in participants’ level of flexibility over time. In general, this pilot study will inform a larger trial that will significantly add to the literature on the use of CRT with adolescents with AN.

## Abbreviations

AN: Anorexia nervosa
CHOP: Children’s Hospital of Philadelphia
CRT: Cognitive remediation therapy
FFT: Family Fun Time
IRB: Institutional Review Board
LGCM: Latent Growth Curve Modeling
TAU: Treatment as usual
TTP: Teach the Parent

## Footnotes

1 The inclusion criteria include those who have subthreshold AN at recruitment or enrollment. In our experience, adolescents will often not report any elements of Criterion B (fear of gaining weight, becoming fat, or engagement in behavior that interferes with weight gain) until nutritional rehabilitation has begun. Thus, it may take a number of days of nutritional rehabilitation for strict DSM-5 criteria to be met. In order to capture adolescents within in the first days of hospitalization, we include those with sub-threshold AN. Many adolescents who are recruited with this symptom profile will be diagnosed with AN upon discharge. Because of this, we refer to participants as being diagnosed with AN throughout the protocol. When we report the findings from this study, we will include diagnosis at enrollment and diagnosis at discharge in order to accurately represent the sample.
